# The Collaborative African Genomics Network (CAfGEN): Applying Genomic technologies to probe host factors important to the progression of HIV and HIV-tuberculosis infection in sub-Saharan Africa

**DOI:** 10.12688/aasopenres.12832.2

**Published:** 2018-06-21

**Authors:** Gerald Mboowa, Savannah Mwesigwa, Eric Katagirya, Gaone Retshabile, Busisiwe C. Mlotshwa, Lesedi Williams, Adeodata Kekitiinwa, David Kateete, Eddie Wampande, Misaki Wayengera, Betty Nsangi Kintu, Grace P. Kisitu, Samuel Kyobe, Chester W. Brown, Neil A. Hanchard, Graeme Mardon, Moses Joloba, Gabriel Anabwani, Ed Pettitt, Masego Tsimako-Johnstone, Ishmael Kasvosve, Koketso Maplanka, Sununguko W. Mpoloka, Makhosazana Hlatshwayo, Mogomotsi Matshaba

**Affiliations:** 1 Department of Immunology and Molecular Biology, College of Health Sciences, Makerere University, Kampala, Uganda; 2 Department of Medical Microbiology, College of Health Sciences, Makerere University, Kampala, Uganda; 3 Department of Biological Sciences, Faculty of Sciences, University of Botswana, Gaborone, Botswana; 4 Baylor College of Medicine Children's Foundation-Uganda, Kampala, Uganda; 5 Department of Bio-molecular Resources, College of Veterinary Medicine, Makerere University, Kampala, Uganda; 6 Genetics Division, Department of Pediatrics , University of Tennessee Health Science Center, Memphis, Memphis, TN, USA; 7 Le Bonheur Children's Hospital, Memphis, Memphis, TN, USA; 8 St. Jude Children's Research Hospital, Memphis, TN, USA; 9 Department of Molecular and Human Genetics, Baylor College of Medicine, Houston, TX, USA; 10 Department of Pediatrics, Baylor College of Medicine, Houston, TX, USA; 11 ARS/USDA/Children's Nutrition Research Center, Department of Pediatrics, Baylor College of Medicine , Houston, TX, USA; 12 Department of Pathology and Immunology, Baylor College of Medicine, Houston, TX, USA; 13 Botswana-Baylor Children's Clinical Centre of Excellence, Gaborone, Botswana; 14 Baylor College of Medicine Children's Foundation-Swaziland, Mbabane, Swaziland; 15 Department of Medical Laboratory Sciences, University of Botswana, Gaborone, Botswana; 16 Pediatric Retrovirology, Department of Pediatrics, Baylor College of Medicine, Houston, TX, USA

**Keywords:** Bioinformatics, Genetics, Genomics, HIV/AIDS, Pediatrics, Tuberculosis, Education, Development

## Abstract

**Background**: Here, we describe how the Collaborative African Genomics Network ( *CAfGEN)* of the Human Heredity and Health in Africa (H3Africa) consortium is using genomics to probe host genetic factors important to the progression of HIV and HIV-tuberculosis (TB) coinfection in sub-Saharan Africa.   The H3Africa was conceived to facilitate the application of genomics technologies to improve health across Africa..

**Methods**: *CAfGEN* is an H3Africa collaborative centre comprising expertise from the University of Botswana; Makerere University; Baylor College of Medicine Children’s Clinical Centers of Excellence (COEs) in Botswana, Uganda, and Swaziland; as well as Baylor College of Medicine, Texas. The COEs provide clinical expertise for community engagement, participant recruitment and sample collection while the three University settings facilitate processing and management of genomic samples and provide infrastructure and training opportunities to sustain genomics research.

**Results**: The project has focused on utilizing whole-exome sequencing to identify genetic variants contributing to extreme HIV disease progression phenotypes in children, as well as RNA sequencing and integrated genomics to identify host genetic factors associated with TB disease progression among HIV-positive children. These cohorts, developed using the COEs’ electronic medical records, are exceptionally well-phenotyped and present an unprecedented opportunity to assess genetic factors in individuals whose HIV was acquired by a different route than their adult counterparts in the context of a unique clinical course and disease pathophysiology.

**Conclusions**: Our approach offers the prospect of developing a critical mass of well-trained, highly-skilled, continent-based African genomic scientists. To ensure long term genomics research sustainability in Africa, *CAfGEN *contributes to a wide range of genomics capacity and infrastructure development on the continent, has laid a foundation for genomics graduate programs at its institutions, and continues to actively promote genomics research through innovative forms of community engagement brokered by partnerships with governments and academia to support genomics policy formulation.

## Introduction

In 2011, a group of scientists came together and conceived the white paper “ Harnessing Genomic Technologies Toward Improving Health in Africa: Opportunities and Challenges” ^1^. Currently, much research in Africa is funded through foreign granting mechanisms ^2^ and many PhD level scientists in Africa have received their training at foreign institutions ^3^. Unfortunately, scientists often leave for training and do not return to Africa because of limited opportunities on the continent ^4^. The Human Heredity and Health in Africa consortium (H3Africa ) was initiated, in part, to reverse this ‘brain drain’ and has already made substantial strides towards this goal and the Collaborative African Genomics Network ( *CAfGEN*) is an H3Africa collaborative centre.

The clinically important research goals of *CAfGEN* are providing us new insight into the pathophysiology of paediatric HIV and HIV-TB in sub-Saharan Africa (SSA) in order to ultimately facilitate development of new treatment strategies. *CAfGEN* continues to support sustainable genetic research in Africa to overcome the dual deficiencies of too little genomics expertise and infrastructure. Subsequently, all *CAfGEN* research projects are deliberately designed to impart transferable expertise that stretches across the breadth of genetic and genomics studies - study design, Ethical, Legal and Social Implications (ELSI), informed consent and sample collection, sample processing and storage, data generation and bioinformatics, statistical analysis of association studies, and reporting and publication of results. *CAfGEN* researchers and clinicians have a solid understanding of how the rapidly changing world of genomics can and should be applied to answer the most pressing questions in the African settings.

The completion of the Human Genome Project in 2003 catapulted efforts to use genomics to enhance our understanding of human diseases ^5^. Key to this, is identifying and utilizing variation in the human genome that is associated with disease-specific onset, complications, or outcomes. Some inherited diseases, such as Sickle cell disease (SCD), are single gene or Mendelian disorders arise as a result of mutations in one or both alleles of a gene while others, such as Chronic Kidney Disease, are the result of a complex interplay of factors that can include a multitude of both genetic and environmental influences, acting to both increase or decrease susceptibility. Added to this are circumstances where there is inter-individual variation in infection susceptibility or resistance ^6, 7^ or severity of infection ^6– 8^, and host genetics can also play a major role in determining the most effective treatment for diseases such as cancer or predicting adverse reactions to certain drugs ^9^.

Genetics and genomics have a particularly important role to play in biomedical research as DNA variation can directly affect disease outcomes ^10, 11^ leading to variations in disease susceptibility, resistance, and rate of progression. Understanding this variation, therefore, has the potential to lead to a better understanding of the biology, physiology, and chemical pathways involved in disease and can help direct scientists to new areas of research that may result in better medications and treatment options. To date, this approach has yielded several pharmacogenomic biomarkers that have been translated into clinical practice, affecting the use of medications to improve quality of life for individuals on specific treatments ^12^. Reflecting on the safety of antiretroviral medications; in SSA, efavirenz forms the preferred first-line anti-retroviral therapy for those over the age of 3 years but adverse drug events have been reported. Additionally, some HIV-infected individuals of African origin are predisposed to developing adverse efavirenz-induced neuropsychiatric responses due to variants in *CYP2B6* that impair normal metabolism of efavirenz ^13^. The CYP2B6*6 allele occurs at a high frequency in people of African origin and is associated with high efavirenz concentrations and genotype-screening is recommended in these populations ^14^. Genomic screening in populations has also identified the HLA-B*5701 variant that predicts Abacavir hypersensitivity; assays for this variant are now routinely performed ahead of time to minimize drug associated reactions in the Western world ^12, 15^. Africa is the evolutionary home of modern humans; and thus, comprises the greatest human genetic diversity among major world populations ^10– 12^. In addition, Africa hosts a wide variety of pathogens, climates, lifestyles, and habitats, some of which are unique to the continent. These observations underscore the immense potential for learning more about human health and our environment through systematic study of the interplay of genetics and environment in African populations ^11^.

## Genesis of the Collaborative African Genomics Network ( *CAfGEN*)

After the H3Africa white paper, a request for application (RFA) was released and collaborative centers applied for funding in health-related areas of research (see NIH H3Africa grant page), including ;

1. The genetic/environmental contributors to non-communicable disease in Africa
2. The genetic/environmental contributors to communicable disease in Africa
3. The contribution of the human microbiome to health and disease in Africa
4. Mendelian diseases in Africa
5. Pharmacogenomics
6. AIDS and co-morbidities
7. Genetic and genomic basis of HIV/AIDS disease, abuse of licit or illicit substances (including alcohol, tobacco, cannabis, stimulants, opiates), and related co-infections or co-morbidities.

This call proved to be the genesis for the formation of the Collaborative African Genomics Network - *CAfGEN*. *CAfGEN* aims to redress the scientific imbalance of genomics research of African paediatric populations. Our network incorporates six sites – the Botswana, Uganda and Swaziland Children’s Clinical Centres of Excellence (COEs); the University of Botswana; Makerere University; and Baylor College of Medicine in Houston, Texas. The *CAfGEN* research agenda includes:

1. Recruitment of prospective and retrospective cohorts of HIV and HIV-TB infected children;
2. Development of core genomics facilities within Africa for sample processing and storage;
3. Candidate gene re-sequencing, HLA allelotyping and whole-exome sequencing (WES) of patients at the extremes of HIV disease progression;
4. Integrated genomic analyses of active TB progression and associated clinical outcomes using expression quantitative trait loci (eQTL) analysis.

These projects are being undertaken in the context of an extensive training and career development plan that will also see significant upgrades in African human capital and genomics infrastructure ^16^. In so doing, *CAfGEN* creates a unique, highly synergistic African alliance that is contributing novel and important mechanistic insights to paediatric HIV and HIV-TB disease progression ^17^ while establishing sustainable genomics technology, expertise, and capacity on the African continent.

## A genomics development framework for 21 ^st^ century Africa

*CAfGEN* seeks “to create a collaborative, multi-disciplinary, multi-institutional, inter- and intra-country network of African scientists, clinicians, and researchers using genomics approaches to study gene/environment interactions for HIV/AIDS, its co-morbidities, and other diseases among diverse paediatric African populations.” To meet the attendant challenges of accomplishing this mission, a highly collaborative, synergistic, network of institutions has been assembled.

Firstly, the project draws upon the extensive clinical and research experience of the Botswana, Swaziland and Uganda COEs - together the three COEs have >35 years of experience providing state-of-the-art care and treatment of paediatric HIV that extends to >20,000 HIV-infected children across Botswana, Swaziland and Uganda. The COEs are headquarters for countrywide research related to the care, treatment, and prevention of paediatric HIV/AIDS and co-occurring diseases and are affiliated with the Baylor International Pediatric AIDS Initiative (BIPAI) - a state-of the-art paediatric HIV/AIDS health care network that spans across 11 African nations, including major centres in Malawi, Lesotho, and Tanzania, which have expressed strong interest in collaborating with *CAfGEN*. The COEs are the clinical backbone of *CAfGEN*; their expertise substantially mitigates the challenges of proper phenotyping and sample numbers needed for large-scale genomics.

To this backbone, we appended molecular genetics expertise at two universities that are closely (geographically and academically) related to the COEs: Makerere University, Uganda, one of the most prestigious academic universities in East Africa ^18^, has extensive experience with infectious disease and genetic studies, including *Mycobacterium tuberculosis* and HIV; and the University of Botswana (UB), which has a growing expertise in human genetics and significant monetary and human investment in molecular genetics, bolstered by a multi-million dollar Medical Education Partnership Initiative (MEPI) grant, with which *CAfGEN* shared a Principal Investigator.

To further develop the genetic and genomic capabilities of the network, we partnered with Baylor College of Medicine in Houston, Texas, to afford trainees access to high-level genomics expertise, including hands-on laboratory experiences, didactic coursework, data analysis, and a variety of other educational activities ^16^. These activities are then transferred to the home African countries and become rooted in the provision of new faculty with high-level training and expertise – North-South collaboration for the 21 ^st^ century.

**Figure 1.**
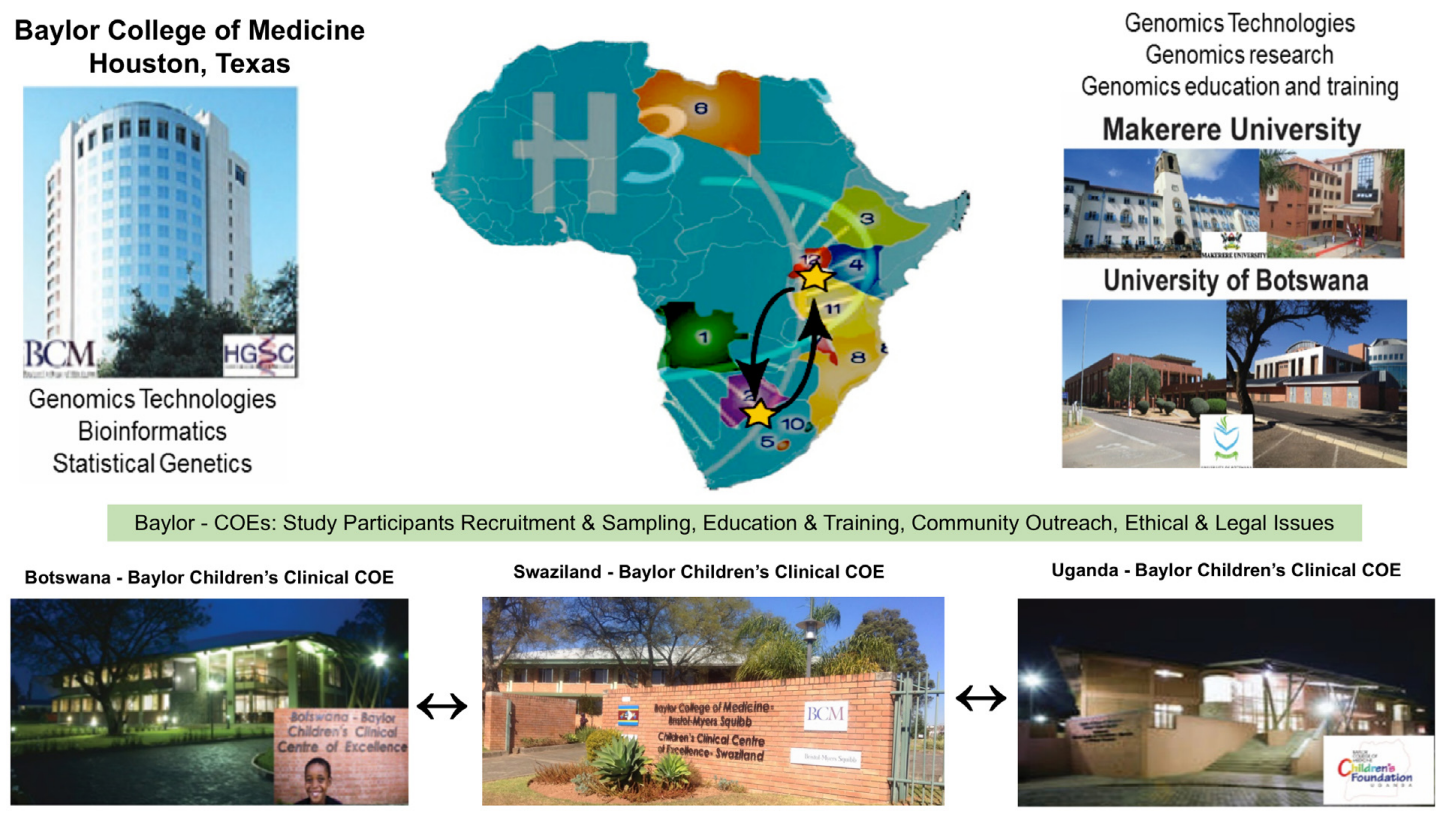
*CAfGEN* collaborating institutions.

The culmination of these centres, as shown in Figure 1 below, is the genesis for a unique African alliance using a highly synergistic approach to contribute novel and important mechanistic insights to disease progression in paediatric HIV and HIV-TB co-infection.

## Specific aims of *CAfGEN*

Disproportionately few advanced genetic and genomic studies have involved the indigenous peoples of Africa, and even fewer have included African paediatric populations ^19– 21^. Yet, paradoxically, these populations collectively carry a large proportion of the human disease burden that results in significant mortality and morbidity. HIV/AIDS and TB – HIV’s most frequent co-morbidity – exemplify this scientific disparity; together, HIV and HIV-TB result in more than 500,000 new childhood cases every year (see UN report on combatting HIV/AIDS, Malaria and Other Diseases). Studies of host genetic factors underlying Long-Term Non-Progressors (LTNPs) of HIV infection have led to new therapies through the identification of loci that are important to *in vivo* control of virus pathogenicity ^22^. Similarly, a detailed understanding of the temporal *in vivo* host molecular events occurring in the progression to active TB disease in the face of HIV co-infection, could significantly impact development of effective therapeutic strategies. Although genomic approaches have been used to identify host response pathways that are important to TB-disease progression, almost all of these studies were undertaken in non-African, adult populations. HIV-infected Africans and particularly children - who have a different route of acquisition, clinical course, and pathophysiology from their adult counterparts - have not been included, although they potentially have more to ultimately contribute and gain from any therapeutic advances. *CAfGEN* envisaged a project that encompassed 5 inter-related aims:

*Aim 1*: Recruit a cohort of well-phenotyped paediatric HIV and HIV-TB infected patients. Retrospectively recruit 500 LTNPs and 500 rapid progressors (RPs) controls through the Electronic Medical Record (EMR).
*Aim 2*: Create a DNA and RNA bioarchive of HIV- and HIV-TB infected paediatric patients from blood and sputa
*Aim 3*: Evaluate the role of established HIV disease progression susceptibility loci in paediatric HIV by undertaking gene sequencing and allelotyping of candidate loci in RP and LTNP patients.
*Aim 4*: Identify novel host alleles influencing paediatric HIV disease progression by conducting whole-exome sequencing to identify variants that are associated with either RPs or LTNP.
*Aim 5*: Identify genes that show significant temporal differential expression with progression to active TB disease by performing RNA sequencing of paired samples taken from HIV co-infected children at baseline and again at the time of active TB disease progression.
*Aim 6*: Identify genes key to the progression to active TB by combining differential gene expression with SNP genotyping to identify expression quantitative trait loci (eQTL) and performing integrated genomic analyses of active TB disease and related clinical outcomes.
*Aim 7*: Establish and enhance undergraduate, graduate, and faculty education in genetics/genomics and provide opportunities for long- and short-term training of scientists and technicians from African universities.
*Aim 8*: Establish genetic and genomic technologies and supporting laboratory and physical infrastructure for large-scale genetic/genomic analyses of common diseases in Africa.

These strategic aims were designed to create independent, sustainable genomics facilities in Africa that recruit and train new scientists in genomics, who can then apply these technologies to solve widespread relevant problems in human diseases in Africa.

## Methods

### Infrastructure development

**Figure 2.**
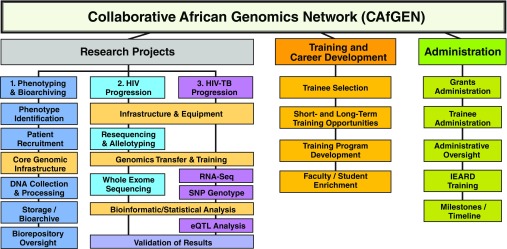
*CAfGEN* Activities.

The clinically important research goals of *CAfGEN* are set to uncover specific genetic markers and genes that reflect the greatest risk for progression in HIV disease and to gain new insights into the pathophysiology of HIV and HIV-TB coinfection, in order to ultimately facilitate development of new treatment strategies. The current lack of substantial work in these areas in the two main *CAfGEN* countries reflects a general maxim underlying the lack of sustainable genetic research in Africa - the dual deficiencies of too little expertise and supporting infrastructure. In keeping with the mission and vision of H3Africa, the *CAfGEN* approach to its primary research goals framed each project in the context of an opportunity to build sustainable genomics capacity and develop genomics infrastructure. Consequently, all *CAfGEN* research projects are interrelated and are deliberately designed to impart transferable expertise that stretches across the breadth of genetic and genomics studies: study design, Ethical Legal and Social Issues (ELSI), consent and sample collection, sample processing and storage, data generation and bioinformatics, statistical analysis of association studies and publication of results ( *Figure 2: illustrates the core branches of the network*). Further, each project has a tangible upgrade in physical infrastructure and capacity. At the end of the grant period, *CAfGEN* researchers and clinicians will have been equipped with a solid understanding of how the rapidly changing world of genomics can be applied to answer the most pressing questions on African continent. Moreover, they are receiving the necessary equipment and facilities to design and implement future genomics studies in a culturally sensitive, sustainable fashion that engenders public trust, acceptance and avoids the pitfalls of some genomic research models of the past. The administrative personnel were trained in grants management and oversight equipping them with skills to administer large, multicenter research grants.

### Ethics and informed consent

Each participating institution had to undergo ethical review processes by the respective institutional review boards (IRBs), followed by the submission of the research protocol by the main applying institution, being the Botswana-Baylor Children’s Clinical Centre of Excellence in Gaborone, to the Botswana Ministry of Health Research and Development Committee for ethical approval. IRB approvals were obtained for all participating institutions.

Written informed consent was sought from the participant and/or their parents or legal guardians. Consent forms were prepared in both English and the main local language at each study site (Luganda in Uganda, Setwana in Botswana, and Swati in Swaziland). The parent or legal guardian provided written informed consent on behalf of participants who were younger than the legal age of consent (18 years in Uganda, Swaziland and 21 years in Botswana). Those who were older than the legal age of consent signed for themselves. Assent forms were prepared for children and adolescents aged between 12 and the legal age of consent (in addition to their parents/legal guardians’ written informed consents). All subjects were free to decline participation in the study and it was made clear that non-participation would not affect any aspect of medical care they were receiving.

While HIV infection and its comorbidities remain the central foci of the project, the broader *CAfGEN* goal is to empower new investigators with a wide array of skills that are required to examine any clinical problem that would be suitable for a genomics approach. We envisioned a critical mass of well-trained, highly knowledgeable, African human geneticists and genomicists each with a broad spectrum of expertise, the knowledge and support to secure outside funding, the ability to educate future generations of researchers, and the experience to collaboratively empower clinicians and researchers in a multi-disciplinary manner.

### Identification, selection and training of CAfGEN trainees

During the first award period of the grant (2014 to 2016), six trainees were identified - three each from Botswana and Uganda. Trainees were recruited through the University of Botswana and Makerere University via both internal and external advertising. Selection criteria included a postgraduate science degree, with preference given to those in the fields of genetics, cell biology, biostatistics, and computer science ^16^. Candidates who were short-listed based on their academic background and an essay describing their motivation, as well as testimonials provided by three of their academic referees, were subsequently invited for oral interviews conducted by a panel of university staff. Potential scholarship recipients identified at this stage were required to successfully register with the university as PhD students and submit a research proposal based on the *CAfGEN* research objectives.

The selection process took approximately three months at each university. Trainees took graduate genomics and bioinformatics courses, seminars, and workshops that were not available at their home institutions. They also undertook laboratory rotations at selected BCM departments such as the United States Department of Agriculture (USDA)/Agricultural Research Service (ARS) Children’s Nutrition Research Center, Human Genome Sequencing Center, Center for Statistical Genetics, RNA-Sequencing, as well as the Computational and Integrative Biomedical Research Center. In addition, they participated in the annual BCM’s Department of Molecular and Human Genetics sponsored two-day retreats at Galveston, Texas where they each presented their PhD concepts and proposals. The trainees have published a peer-reviewed report detailing their perspective on training the next generation of African genomic scientists ^16^. All the six students successfully completed more than two years of training at BCM and returned to their respective universities to continue with their PhD studies. Their home universities will assimilate them into genomics faculty positions on completion of their PhDs.

The two participating African universities have enthusiastically embraced genomics training fulfilling *CAfGEN*’s long-term objectives. Tangible strides to nurture the era of genomics at the collaborating institutions have been realized already such as the inauguration of a new department of Immunology and Molecular biology at Makerere University; this is a home for the genomics and bioinformatics graduate programs for which *CAfGEN* laid the foundation stone.

### Recruitment of study participants

Initially, the COEs in Botswana and Uganda were involved in the study participant recruitment. A cohort of well-phenotyped paediatric HIV and HIV-TB infected patients was recruited retrospectively through the EMR, and consisted of 500 LTNPs and 500 RPs. In addition, we recruited HIV-positive children who also progressed to active TB disease. Swaziland COE was added later on to boost the enrolment in the TB case-control component of the study. Blood samples were drawn from the participants and sent to the two universities for the preparation of DNA and RNA.

### Power considerations and replication of results

The *CAfGEN* study is divided into discovery and replication phases. To provide a broad approximation of the power of our study, we made a number of assumptions: a) variants with estimated minor allele frequencies > 0.05; b) an alpha (α) value for statistical significance of *p* < 0.01; c) a genetic relative risk (GRR) of between two and four for LTNP-associated variants d) a log additive disease model. Under these assumptions and using the methods of Gauderman implemented in Quanto 1.2.4, where a sample size of 200 cases and 200 controls had greater than 80% power to detect significant differences between groups at *β* = 0.80 discovery) of the study. Variants meeting an association threshold of *p* < 0.01 are carried forward for targeted allele typing in 300 LTNPs and 300 RPs in the replication phase.

### Community engagement

Community engagement is a key requirement for the overall H3Africa goal of long-term sustainability and has been a prominent feature of the *CAfGEN* plan. We have used a variety of training and educational programs in the form workshops and laboratory training as described below.

*Workshops*: A variety of practical workshops were carried out by the COEs to provide training to individuals within and outside the *CAfGEN* network. The workshops were administered by the COEs including specific expertise such as; a) sputum induction in the children, b) Laboratory procedures for HIV testing, c) collection and processing of whole blood samples for research studies, d) effective use of EMR for research studies, and e) effective counselling to maximize patient compliance and research study participation.

**Figure 3.**
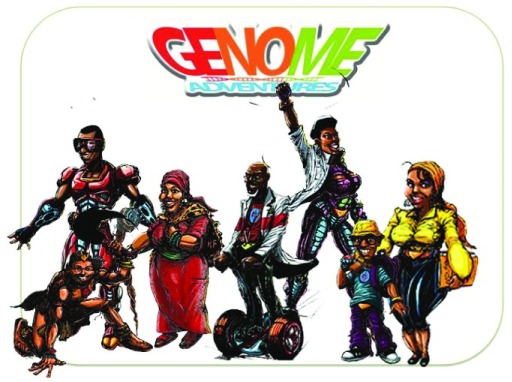
Genome adventures comics.

Other innovative community engagement activities included the production of Genome Adventures comic books. In these series; Kitso, a young man takes a journey with the superheroes to explore the relations of heredity and genetics as shown in Figure 3 below. These series featured on a wide range of social media platforms such as Weebly, Facebook, Twitter, and Pinterest. These books have been translated into several languages such as Setswana, Swahili, Luganda, Arabic, Hausa, French, and Portuguese. This project was funded jointly by the Wellcome Trust and the US National Institutes of Health initiatives.

We also shared close working relations with the Botswana Harvard AIDS Institute Partnership (BHP), a world-renowned institution of excellence in research and education pertinent to HIV/AIDS and other emerging public health challenges. The more than 20 years of collaborative research and training experience at BHP was highly valuable to *CAfGEN.* Their research areas include virology, molecular biology, immunology, human genetics, epidemiology, and social and behavioral issues relevant to the AIDS epidemic in Botswana and southern Africa. We further maintained routine interactions with the community leaders and institutional review boards and community leaders through community advisory boards made up of local community leaders.

### Genomic sample processing, bioarchiving, and shipping

Uniquely labeled blood samples were collected using PAXgene Blood DNA and RNA tubes at the COEs and transported to both the University of Botswana (Department of Biological Sciences, Faculty of Sciences) and Makerere University (Molecular Diagnostics laboratory, Department of Immunology and Molecular Biology) where genomic DNA and RNA was extracted. The team utilized the already existing molecular genetics expertise at these two universities to successfully perform extraction of high quality DNA and RNA for subsequent sequencing and genotyping. Samples were quantified using either a Nanodrop 2000 spectrophotometer or a Qubit 2.0 Fluorimeter. Sample quality was assessed by agarose gel electrophoresis. Samples were stored in a -80°C freezer at the H3Africa Integrated Biorepository at Makerere University. Genomic DNA and RNA were shipped, as per the requirements of the Centers for Diseases Control and Prevention (CDC) and the International Air Transport Association (IATA), to the Human Genome Sequencing Center and the Children's Nutrition Research Center at BCM for WES and RNA sequencing, respectively. Aliquots of all samples are stored at the Biorepository at Makerere University.

## Genomic data analyses and accomplishments

Following the completion of more than two years of didactic training in genomics and bioinformatics at BCM, *CAfGEN* trainees are currently analyzing the genomic data at their respective institutions utilizing local computing resources made possible by *CAfGEN* funding while they continue to receive both mentorship and supervision from their BCM mentors.

Trainees have focused on WES data analysis as part of their PhD requirement at the Universities in Uganda and Botswana. Analysis has revealed uncaptured genetic variation and distinct ancestry in the southern African population of Botswana ^23^. Trainees contributed to a number of studies such as “Whole-exome sequencing of SCD patients with hyperhemolysis syndrome (HHS) suggests a role for rare variation in disease predisposition”. This study highlights a potential role for rare genetic defects in the development of HHS among adult SCD patients ^24^, a disease important to African populations. This work was funded by the US National Blood Foundation and National Human Genome Research Institute. Trainees continue to analyse *CAfGEN* genomic data to answer their respective study objectives including roles of chemokines & their ligands variant in HIV/AIDS disease progression from which the preliminary results suggest no role for chemokines and their cognate ligands in pediatric AIDS disease progression (unpublished data), interrogation of unmapped reads, rare and common variants in paediatric HIV/AIDS disease progression as well as RNA-Seq data analyses.

*CAfGEN* trainees at Makerere University coordinated H3ABioNet‘s Introduction to Bioinformatics three months’ on-line course. This course provides an introduction to the field of bioinformatics, with a focus on important bioinformatics tools and resources. The course attracted 56 participants and this was the first time it was offered at the University. *CAfGEN* trainees have made both oral and poster presentations during the International Society for Computational Biology (ISCB) and African Society for Bioinformatics and Computational Biology (ASBCB) at Entebbe, Uganda; from October 10 – 13, 2017 ^25^.

The trainees have also cultivated collaborations and networks that are important for sustaining and development of genomics capacity in Africa. These include; Bridging Biobanking and Biomedical Research across Europe and Africa (B3Africa), this collaboration enabled Makerere University to receive eB3Kit -mini-computational server. The eB3Kit consists of 3 main components: A bioinformatics platform, an ethical and legal framework, a training component. This server has been very useful in the genomics trainings/workshops held at Makerere. It provides a linux environment which is remotely utilized by workshop participants. *CAfGEN* trainees have been able to apply for collaborative funding opportunities and awarded a fellowship by the THRiVE Consortium (Training Health Researchers into Vocational Excellence in East Africa) also funded by the Wellcome Trust. The support covers the trainee’s PhD related activities and six months of training in the department of Genetics at the University of Cambridge, in the UK.

Other networks that have been established by the trainees include the Makerere University – Uganda Virus Research Institute (UVRI) Centre of Excellence for Infection and Immunity Research and Training ( *MUII-Plus*). The MUII is passionate about the development of bioinformatics in Uganda. They have offered both bioinformatics grants and travel scholarships to the trainees to present their PhD work during the ISCB/ASCB at Entebbe in October, 2017. Trainees are also taking part in grant application processes at their home institutions. This is critical for sustainability of the genomics milestones attained through *CAfGEN* funding; trainees also continue to assist in external sequencing projects and genomic data analyses at their home institutions.

To develop pedagogical skills, trainees are equally involved in teaching undergraduate courses such as introduction to bioinformatics, fundamental genetics & molecular biology for non-medical students and introduction to bioinformatics and medical genomics for MBChB (medical) students. This is an important strategy to ensure trainees retention as faculty as well as knowledge and skills transfer.

## Capacity building and infrastructure development

The *CAfGEN* project has enabled six PhD students to successfully complete their intensive genomics training at BCM. They are all being supported to complete their PhDs at their home institutions. The project allowed the purchase of both an ABI 3500 Capillary sequencer (Life Technologies, CA) and a MiSeq System (Illumina, San Diego, CA) for the University of Botswana and Makerere University, respectively. The acquisition of these instruments has already spurred new opportunities for genomics training and research at these institutions. In particular, the " Introduction to Bioinformatics and Next Generation Sequencing Techniques" workshop was funded by the World Bank through the African Higher Education Centres of Excellence Project (MAPRONANO ACE II) and attended by 52 researchers from Rwanda, Tanzania, Zambia, Ethiopia, Kenya, Malawi, and Uganda, the Bioinformatics RNA sequence data analysis workshop was funded by the Alliance for Global Health Science also attended by 19 participants from Uganda and Zimbabwe and one-year training program funded through the University of Georgia’s “Computational and Molecular Epidemiology in TB and HIV in Uganda”. This mentorship program offers a unique training arrangement to students who have special interest in genomics and bioinformatics to attend weekly tutorials and practical sessions offered by faculty genomics mentors throughout the year. The program is currently training four students at Makerere University (D43TW010045-03). *CAfGEN* trainees are at the centre of these trainings and workshops.

Classroom training was also carried out as part of capacity building such as the “Ethics in Research and Informed Consent: emphasis on African populations”. This lecture targeted health care professionals who were actively engaged with recruited patients for genomics research studies. Special emphasis was given to the challenges that confront carrying out such studies in African populations including informed consent and assent, stigmatization, and effectively conveying the potential impact of such research on the current and future health. This course has been included in the new genomics graduate programs at Makerere University. The importance of genomics to human health (community outreach). This lecture targeted community lay persons and emphasized basic concepts of heredity, genetics, and genomics, common community relevant human genetic conditions (e.g., albinism and Sickle cell disease), and the importance of genomics research to human health and research participants’ protections.

The *CAfGEN* project has also procured and installed a server for bioinformatics at the Makerere University College of Health Sciences. The server is being used by students in the genomics mentorship programs and will soon be utilized by the genomics graduate programs at Makerere University. *CAfGEN* has already fostered many endeavors that are sustainable, productive, impactful, and transformative in both the satellite countries and the continent at large in constructing strong networks and strategies to achieve the goals of the H3Africa initiative. In addition to the above, other highlights include;

1. To ensure long-term, high-quality, sustainable human genetic and genomics studies in Africa, one of our principal investigators (Dr. Moses Joloba) received funding and set up the largest biorepository in East Africa, the Integrated Biorepository of H3Africa Uganda - IBRH3AU.

2. To ensure best practices governing *CAfGEN* and future genomics studies in Africa, our center at Makerere University was awarded an NIH U01 grant entitled “Ethical and social issues in informed consent processes in African genomic research funding (Principal investigator: Dr. Erisa Sabakaki Mwaka, Makerere University College of Health Sciences, Uganda). This project aims to increase awareness and protection of the interests of research participants regarding ELSI (U01-HG009810-01).

3. The "Nurturing Genomics and Bioinformatics Research Capacity in Africa (BRecA)" program, which builds on the momentum of *CAfGEN*, was awarded to Dr. Kateete David and Dr. Graeme Mardon by the Fogarty International Center to establish sustainable genomics and bioinformatics graduate programs at Makerere University (U2RTW010672). Both masters and PhD graduate programs in Genomics and Bioinformatics are in the final stages of approval at Makerere. *CAfGEN* alumni are also highly active in these training programs.

4. *CAfGEN* trainees continue to be outstanding in so many ways including winning travel scholarship awards for both oral and poster presentations at different H3Africa consortium meetings, trainings, and workshops, in 2017 during this 10 ^th^ H3A consortium meeting, a *CAfGEN* trainee excelled and was awarded the prize for the best poster presenter. Another trainee made both an oral and poster presentation during the ISCB/ASCB conference. They have presented their work at several other conferences such as the UCSF-Gladstone - Centers for AIDS Research (CFAR) 9 ^th^ East Africa Collaborative Scientific Symposium at the Infectious Diseases Institute in Kampala, Uganda on the 19 – 20 ^th^ of January 2017. At the end of their training, we envisage a team of African human geneticists and genomicists who have a broad spectrum of expertise and knowledge that will enable them compete for and secure outside funding while they continue the cycle of educating future generations of researchers.

Capacity building and research activities undertaken by CAfGEN at the Swaziland COE included training the investigators in implementation of pediatric tuberculosis intensive case finding among the HIV infected children and held genomics community outreach workshops.

### Challenges encountered and opportunities

Implementing the *CAfGEN* project has not been without challenges, through which we have learnt valuable lessons. Retention of personnel has continued to be a problem at all levels of academia and *CAfGEN* has been no different as some of the *CAfGEN* personnel have since moved on to more lucrative job offers; delayed project approvals/renewals have also been a fact of life - the project required ethics approvals to be in place at all the involved institutions, although genomics studies are new territory for any of the local ethics/Institutional Review Boards. The broader H3Africa consortium was faced with similar issues, and this ultimately led to sponsored invitations to Head of Ethics committees/IRBs to attend H3Africa meetings in order to voice their concerns and engage in dialogue to mitigate any grievances. This process has paid significant dividends - green-lighting projects that were on hold for some time, and gaining a wider audience and appreciation for H3Africa protocols at the University and Ministry levels. Finally, communication and data transfer issues due to slow or absent internet connections continue to be a significant problem without an obvious solution thus far. Genomics generates large amounts of data, necessitating fast connections for analyses and data transfer; although increased bandwidth and speed are a priority for many African governments, progress on this front has been slow.

Other challenges of pediatric HIV/AIDS and TB genomics research included;

a) Immature immune system that prompts HIV/AIDS to develop more rapidly than in their adult counterparts
b) Technical expertise in HIV testing algorithm for infants born to HIV- positive women includes HIV RNA/DNA - polymerase chain reaction (PCR) test which is more technically demanding and has a relatively longer turnaround time for HIV diagnosis unlike the routine rapid immunochromatographic screening HIV test for detection of antibodies to HIV-1
c) Adherence to antiretroviral therapy and retention for HIV-infected adolescents, social stigma in both communities and schools affecting their school performance, health professionals required to care for HIV-infected children are in short supply generally in resource limited settings
d) New policies such as HIV 'test and treat' strategy and isoniazid prophylactic therapy for the prevention of tuberculosis in HIV-infected children
e) The challenges of TB case finding amongst HIV infected children.

CAfGEN and the COEs addressed some of the above challenges. The COEs perform HIV RNA PCR routine testing for infants born to HIV-infected women and ensure a short turnaround time for this diagnosis. The COEs have both nationally and internationally well–trained healthcare professionals who manage their ART and TB clinics, ensure adherence to ART treatment, retention for HIV-infected children, and psychosocial well being of the children.

### Dissemination of findings

The findings of the *CAfGEN* project will be disseminated in open access peer reviewed journals as required by H3Africa publications policy. All data will be made available to the research community upon request as per the H3Africa Consortium Data Sharing, Access and Release (DSAR) Policy.

### Study status

The *CAfGEN* trainees are currently analyzing the genomic data (exome and RNA-Seq) as well as writing manuscripts as part of their PhD requirements at African institutions.

## Conclusions

The opportunity offered by studying HIV and HIV-TB coinfection in children at the COEs and integrated genomics training in both Botswana and Uganda has permitted us to pioneer a new method of undertaking genomics and biomedical research in Africa whilst building sustainable expertise and infrastructure. This innovative approach has registered significant gains in achieving the goal of H3Africa. Furthermore, we have successfully ensured that the acquired genomics expertise is transferred to Africa as trainees returned to their home countries to take faculty positions, transfer skills and participate in genomics-driven research pertinent to African populations with a goal of improving health.

*CAfGEN* will continue to work hand in hand with the African academia, funding agencies, and governments to promote genomics training, research, and support genomics policy formulation, in pursuit of its long-term goal of becoming a major centre for large-scale genomic studies of paediatric HIV and associated co-morbidities in sub-Saharan Africa.

## Data availability

All data underlying the results are available as part of the article and no additional source data are required.
