# Supplementary material for: The Collaborative African Genomics Network (CAfGEN): Applying Genomic technologies to probe host factors important to the progression of HIV and HIV-tuberculosis infection in sub-Saharan Africa
Source: AAS Open Res. 2018 Jun 21;1:3. Originally published 2018 Apr 18. [Version 2] doi: 10.12688/aasopenres.12832.2 (PMC6358002; doi:10.12688/aasopenres.12832.2)
Supplement: Peer review history

# Peer review: Reviewer response for version 2

**DOI:** 10.21956/aasopenres.13951.r26487

**Published:** 2018-06-22

**Authors:** Edward J Hollox

**Affiliations:** 1 Department of Genetics and Genome Biology, University of Leicester, Leicester, UK

No new comments.

I confirm that I have read this submission and believe that I have an appropriate level of expertise to confirm that it is of an acceptable scientific standard.

# Peer review: Reviewer response for version 1

**DOI:** 10.21956/aasopenres.13898.r26408

**Published:** 2018-05-21

**Authors:** Jantina de Vries, Syntia Nchangwi Munung

**Affiliations:** 1 University of Cape Town (UCT), Cape Town, South Africa; 2 Department of Medicine, University of Cape Town (UCT), Cape Town, South Africa

In this paper, Mbwoowa *et al *present the CafGEN project, and describe how it operates, what the pertinent challenges are, and how it has gone about community engagement. The paper illustrates that a well organised and funded collaborative genomics project in Africa can, in a short period of time, achieve a lot in terms of research capacity building. That is quite an encouraging and important message in the manuscript. 

 Major Comments:

 In both the abstract and in the introduction, you first mention H3Africa and then CafGEN, and we would suggest that you reverse that order. In doing that, you’ll give more prominence to your own work – a small but important change of focus. In the way you present your work, we think you should make it clear that CafGEN derives legitimacy from the pertinent scientific questions in this field, and not from H3Africa. H3Africa is the facilitator of CafGEN through funding, network building, and infrastructure – it is not the reason that CafGEN exists though.

- In the same vein, it is not that interesting for an audience to know that ‘a call for applications was issued’. Rather, why don’t you speak about the most important challenges in the field of (paediatric) HIV/AIDS research that motivated you to set up CafGEN? With a follow up section on how CafGEN will systematically tackle those questions.
  - So in short, what we would suggest is that you review your entire first page of the manuscript, and cut out much of the current text, and rather introduce the context pertinent to CafGEN specifically. In a way, we would suggest re-writing your text to demonstrate (rather than state) that CafGEN is a ‘unique, highly synergistic African alliance’
  - One example of that is that it’s only on pg 5 of the current manuscript that you introduce the three Centers of Excellence that CafGEN work is premised on – but isn’t the collective experience in those centres (and a detailed understanding of the knowledge gaps derived thereof) the backbone to the collaboration? If so, then this should take centre stage in the article.
  - In a way, we propose that the text on Pg 6 achieves some of our suggestions above.

 

 Minor comments:

 1. One of the 6 CAFGEN sites is the Swaziland Children’s Clinical Centres of Excellence (Page 4). But the article is silent on research and capacity building activities in this country/site, except that the Swaziland COE was added later on to boost the enrolment in the TB case-control component of the study (see page 8). It therefore appears that much of the capacity building and research activities took place in Uganda and Botswana and not Swaziland. Maybe the authors can add a few sentences on the capacity building and research activities at this site. If none, then maybe explain why that wasn’t the case and the challenges they had in implementing capacity building and research in Swaziland.

 2. Page 4: I think the use of heart disease as an example of an inherited disease may not be the best. Perhaps it is the way it is used in the sentence – we would encourage the authors to review this sentence and the example and to perhaps to use another example or to reword the sentence.

 3. Page 7. Consent forms where translated to Luganda and Setswana. Again, information for the Lesotho site is missing, though there was participant enrolment at the site. Please can you add information on consent, Recruitment/CE for the Lesotho site or otherwise explain how this site fits in with the other sites.

 4. Page 8. Community engagement activities:The CAFGEN comic series is described as an innovative (maybe creative?) CE method (and we agree). The authors can maybe add a sentence or two on why they consider it innovative and also a few lines, in the discussion, on the advantages of this CE method for genomics research in Africa. This will benefit readers who are not familiar with CE in health research in Africa or the use of comics and social media for CE in health research in Africa.

 The first community engagement activity -- classroom training for health care workers may fit better in the section on “Capacity building and infrastructure development”. This is perhaps more an activity concerning training in research ethics for health care workers than a community engagement activity?

We confirm that we have read this submission and believe that we have an appropriate level of expertise to confirm that it is of an acceptable scientific standard.

# Response

**Published:** 2018-06-17

**Authors:** Gerald Mboowa

**Affiliations:** Makerere University, USA

Major Comments:

In both the abstract and in the introduction, you first mention H3Africa and then CAfGEN, and we would suggest that you reverse that order. In doing that, you’ll give more prominence to your own work – a small but important change of focus. In the way you present your work, we think you should make it clear that CAfGEN derives legitimacy from the pertinent scientific questions in this field, and not from H3Africa. H3Africa is the facilitator of CAfGEN through funding, network building, and infrastructure – it is not the reason that CAfGEN exists though.

**We have addressed the order of first mention of “ *CAfGEN” in* the abstract.**

**Here, we describe how the Collaborative African Genomics Network ( *CAfGEN*) of the Human Heredity and Health in Africa (H3Africa) consortium is using genomics to probe host genetic factors important to the progression of HIV and HIV-tuberculosis (TB) coinfection in sub-Saharan Africa.   The H3Africa was conceived to facilitate the application of genomics technologies to improve health across Africa.**

- In the same vein, it is not that interesting for an audience to know that ‘a call for applications was issued’. Rather, why don’t you speak about the most important challenges in the field of (paediatric) HIV/AIDS research that motivated you to set up CAfGEN? With a follow up section on how CAfGEN will systematically tackle those questions.

**We have included a section on Challenges and opportunities of pediatric HIV/AIDS and TB genomics research.**

**Other challenges of pediatric HIV/AIDS and TB genomics research included:**

1. **Immature immune system that prompts HIV/AIDS to develop more rapidly than in their adult counterparts**
2. **Technical expertise in HIV testing algorithm for infants born to HIV- positive women includes HIV RNA/DNA - polymerase chain reaction test which is more technically demanding and has a relatively longer turnaround time for HIV diagnosis unlike the routine rapid immunochromatographic screening HIV test for detection of antibodies to HIV- 1 **
3. **Adherence to antiretroviral therapy and retention for HIV-infected adolescents, social stigma in both communities and schools affecting their school performance, health professionals required to care for HIV-infected children are in short supply generally in resource limited settings **
4. **New policies such as HIV 'test and treat' strategy and isoniazid prophylactic therapy for the prevention of tuberculosis in HIV-infected children  **
5. **The challenges of TB case finding amongst HIV infected children.**

***CAfGEN*** **and the COEs addressed some of the above challenges. The COEs do perform HIV RNA PCR routine testing for infants born to HIV-positive women and ensure a short turnaround time for this diagnosis. The COEs have both nationally and internationally well–trained health professionals who manage the ART and TB clinics, ensure adherence to treatment, retention for HIV-infected children, and psychosocial well being.**

 

- So in short, what we would suggest is that you review your entire first page of the manuscript, and cut out much of the current text, and rather introduce the context pertinent to CAfGEN specifically. In a way, we would suggest re-writing your text to demonstrate (rather than state) that CAfGEN is a ‘unique, highly synergistic African alliance’

 

**We have reviewed the entire first page of the manuscript making it *CAfGEN* pertinent demonstrating that CAfGEN is a ‘unique, highly synergistic African alliance’**

**In 2011, a group of scientists came together and conceived the white paper “Harnessing Genomic Technologies Toward Improving Health in Africa: Opportunities and Challenges” *^1^*. Currently, much research in Africa is funded through foreign granting mechanisms ^*2*^ and many PhD level scientists in Africa have received their training at foreign institutions ^3^. Unfortunately, scientists often leave for training and do not return to Africa because of limited opportunities on the continent *^4^*. The Human Heredity and Health in Africa consortium (H3Africa) was initiated, in part, to reverse this ‘brain drain’ and has already made substantial strides towards this goal and the Collaborative African Genomics Network ( *CAfGEN*) is an H3Africa collaborative centre.**

**The clinically important research goals of *CAfGEN* are providing us new insight into the pathophysiology of paediatric HIV and HIV-TB in SSA in order to ultimately facilitate development of new treatment strategies. *CAfGEN* continues to support sustainable genetic research in Africa to overcome the dual deficiencies of too little genomics expertise and infrastructure. Subsequently, all *CAfGEN* research projects are deliberately designed to impart transferable expertise that stretches across the breadth of genetic and genomics studies – study design, Ethical, Legal and Social Implications (ELSI), informed consent and sample collection, sample processing and storage, data generation and bioinformatics, statistical analysis of association studies, and reporting and publication of results. *CAfGEN* researchers and clinicians have a solid understanding of how the rapidly changing world of genomics can and should be applied to answer the most pressing questions in the African settings.**

- One example of that is that it’s only on pg 5 of the current manuscript that you introduce the three Centers of Excellence that CAfGEN work is premised on – but isn’t the collective experience in those centres (and a detailed understanding of the knowledge gaps derived thereof) the backbone to the collaboration? If so, then this should take centre stage in the article.

**Agreed, we shall be working on a separate manuscript to cover this important aspect of collective experience in COEs also detailing the knowledge gaps and collaboration.**

 

- In a way, we propose that the text on Pg 6 achieves some of our suggestions above.

 

Minor comments:

1. One of the 6 CAfGEN sites is the Swaziland Children’s Clinical Centres of Excellence (Page 4). But the article is silent on research and capacity building activities in this country/site, except that the Swaziland COE was added later on to boost the enrolment in the TB case-control component of the study (see page 8). It therefore appears that much of the capacity building and research activities took place in Uganda and Botswana and not Swaziland. Maybe the authors can add a few sentences on the capacity building and research activities at this site. If none, then maybe explain why that wasn’t the case and the challenges they had in implementing capacity building and research in Swaziland.

**We have added a line regarding the capacity building activities done at the Swaziland COE.**

**Capacity building and research activities undertaken by CAfGEN at the Swaziland COE included training the investigators in implementation of pediatric tuberculosis intensive case finding among the HIV infected children and held genomics community outreach workshops.**

2. Page 4: I think the use of heart disease as an example of an inherited disease may not be the best. Perhaps it is the way it is used in the sentence – we would encourage the authors to review this sentence and the example and to perhaps to use another example or to reword the sentence.

**We have rewritten the sentence as below:**

**Some inherited diseases, such as Sickle cell disease (SCD), are single gene or Mendelian disorders arise as a result of mutations in one or both alleles of a gene while others, such as Chronic Kidney Disease, are the result of a complex interplay of factors that can include a multitude of both genetic and environmental influences, acting to both increase or decrease susceptibility.**

3. Page 7. Consent forms where translated to Luganda and Setswana. Again, information for the Lesotho site is missing, though there was participant enrolment at the site. Please can you add information on consent, Recruitment/CE for the Lesotho site or otherwise explain how this site fits in with the other sites.

**We have included the statement regarding Swaziland informed consent process.**

**Consent forms were prepared in both English and the main local language at each study site (Luganda in Uganda, Setwana in Botswana, and** **Swati** **in Swaziland). The parent or legal guardian provided written informed consent on behalf of participants who were younger than the legal age of consent (18 years in Uganda, Swaziland and 21 years in Botswana).**

4. Page 8. Community engagement activities: The CAfGEN comic series is described as an innovative (maybe creative?) CE method (and we agree). The authors can maybe add a sentence or two on why they consider it innovative and also a few lines, in the discussion, on the advantages of this CE method for genomics research in Africa. This will benefit readers who are not familiar with CE in health research in Africa or the use of comics and social media for CE in health research in Africa.

The first community engagement activity -- classroom training for health care workers may fit better in the section on “Capacity building and infrastructure development”. This is perhaps more an activity concerning training in research ethics for health care workers than a community engagement activity?

**We have moved the classroom training to capacity building section.**

# Response

**Published:** 2018-06-18

**Authors:** Gerald Mboowa

**Affiliations:** Makerere University, USA

One example of that is that it’s only on pg 5 of the current manuscript that you introduce the three Centers of Excellence that CAfGEN work is premised on – but isn’t the collective experience in those centres (and a detailed understanding of the knowledge gaps derived thereof) the backbone to the collaboration? If so, then this should take centre stage in the article.

**Agreed, At the end of the grant period, we shall be working on a separate manuscript to cover this important aspect of collective experience in COEs also detailing the knowledge gaps and collaboration.**

# Peer review: Reviewer response for version 1

**DOI:** 10.21956/aasopenres.13898.r26342

**Published:** 2018-04-30

**Authors:** Edward J Hollox

**Affiliations:** 1 Department of Genetics and Genome Biology, University of Leicester, Leicester, UK

This paper describes the construction and aims of the Collaborative African Genomics Network (CAfGEN), with a focus on the training on a cohort of African genomics researchers, as well as investigating the host genetic factors that influence progression of HIV and HIV-tuberculosis co-infection in sub-Saharan Africa.

 This is a very useful document that describes the development of CafGEN, and provides information to other African genomics partnerships in approaches to build a collaboration that includes training and research. In particular, it highlights infrastructure and community engagement approaches, and identifies roadblocks to this, and potentially other, capacity-building programmes.

 I only have a few minor suggestions that would improve the manuscript.

1. Page 4, paragraph 2. “Key to this, is identifying”. No comma needed.
2. Page 4, paragraph 2. “relatively straightforward variation in a single gene”. This sentence needs to be clarified, reworded, and perhaps this point should be expanded. Sickle cell disease is, as the authors will know, caused by a single variant in a single gene, there are other genetic disease that are caused by different variants in a single gene, and the complexity increases in a continuum to complex multifactorial disease. The sentence seems to suggest that it is either a single gene disease, or a complex disease. It also seems to suggest that heart disease is an inherited disease  – I’m sure this was not the intention of the authors but the sentence can be read this way.
3. Page 4, paragraph 3. “… DNA variation both directly and indirectly impacts disease outcomes”. The previous paragraph discusses how DNA variation can directly affect disease outcomes, but the authors need to be more explicit how DNA variation indirectly affects disease outcomes.
4. Page 4, column 2, paragraph 1. “classically considered to be the wellspring of modern humanity”. This sentence is vague and should be rewritten. In particular, reconsider the use of the term “classically” as I don’t think this is the case, and try to avoid the circularity in this sentence – evidence for Africa being the origin of modern humans comes from the observed high levels of genetic diversity, while this sentence seems to suggest the inference is the other way around.
5. Page 4, column 2, paragraph 2. “After” is clearer than “Subsequent to”.
6. Page 6 column 1. It is not clear why some aims are split into 2 – a and b. Either combine these to make five aims, or give them different numbers to make 8 aims.
7. Page 6, aim 2a. It is not clear why “established” is in scare quotes. Do the authors mean that these loci are established in Europeans but not Africans? The authors should clarify this.
8. Page 7, paragraph 1. The second sentence is not clear. I’d suggest rewording: “The parent or legal guardian provided written informed consent on behalf of participants who were younger than the legal age of consent (18 years in Uganda and 21 years in Botswana).”

I confirm that I have read this submission and believe that I have an appropriate level of expertise to confirm that it is of an acceptable scientific standard.

# Response

**Published:** 2018-06-17

**Authors:** Gerald Mboowa

**Affiliations:** Makerere University, USA

1. Page 4, paragraph 2. “Key to this, is identifying”. No comma needed.

**Comma removed.**

 

1. Page 4, paragraph 2. “relatively straightforward variation in a single gene”. This sentence needs to be clarified, reworded, and perhaps this point should be expanded. Sickle cell disease is, as the authors will know, caused by a single variant in a single gene, there are other genetic disease that are caused by different variants in a single gene, and the complexity increases in a continuum to complex multifactorial disease. The sentence seems to suggest that it is either a single gene disease, or a complex disease. It also seems to suggest that heart disease is an inherited disease – I’m sure this was not the intention of the authors but the sentence can be read this way.

 

**Sentence re-written as below:**

**Some inherited diseases, such as Sickle cell disease (SCD), are single gene or Mendelian disorders arise as a result of mutations in one or both alleles of a gene while others, such as Chronic Kidney Disease, are the result of a complex interplay of factors that can include a multitude of both genetic and environmental influences, acting to both increase or decrease susceptibility.**

1. Page 4, paragraph 3. “… DNA variation both directly and indirectly impacts disease outcomes”. The previous paragraph discusses how DNA variation can directly affect disease outcomes, but the authors need to be more explicit how DNA variation indirectly affects disease outcomes.

**DNA variation can directly affect disease outcomes leading to variations in disease susceptibility, resistance, and rate of progression.**

1. Page 4, column 2, paragraph 1. “classically considered to be the wellspring of modern humanity”. This sentence is vague and should be rewritten. In particular, reconsider the use of the term “classically” as I don’t think this is the case, and try to avoid the circularity in this sentence – evidence for Africa being the origin of modern humans comes from the observed high levels of genetic diversity, while this sentence seems to suggest the inference is the other way around.

**Sentence re-written “Africa is the evolutionary *home* of *modern humans”.***

1. Page 4, column 2, paragraph 2. “After” is clearer than “Subsequent to”.

**“Subsequent to” replaced by “After”.**

1. Page 6 column 1. It is not clear why some aims are split into 2 – a and b. Either combine these to make five aims, or give them different numbers to make 8 aims.

**All aims renumbered 1 to 8.**

1. Page 6, aim 2a. It is not clear why “established” is in scare quotes. Do the authors mean that these loci are established in Europeans but not Africans? The authors should clarify this.

**HIV disease progression susceptibility loci that will be identified in the CAfGEN paediatric HIV infected cohorts. We have removed the scare quotes.**

1. Page 7, paragraph 1. The second sentence is not clear. I’d suggest rewording: “The parent or legal guardian provided written informed consent on behalf of participants who were younger than the legal age of consent (18 years in Uganda and 21 years in Botswana).”

**We have re-worded the sentence as requested.**
